# Complete Dosage Compensation and Sex-Biased Gene Expression in the Moth *Manduca sexta*

**DOI:** 10.1093/gbe/evu035

**Published:** 2014-02-19

**Authors:** Gilbert Smith, Yun-Ru Chen, Gary W. Blissard, Adriana D. Briscoe

**Affiliations:** ^1^Department of Ecology and Evolutionary Biology, University of California, Irvine; ^2^Boyce Thompson Institute for Plant Research at Cornell University

**Keywords:** Lepidoptera, dosage compensation, sex-biased gene expression, phototransduction, olfaction, mushroom body

## Abstract

Sex chromosome dosage compensation balances homogametic sex chromosome expression with autosomal expression in the heterogametic sex, leading to sex chromosome expression parity between the sexes. If compensation is incomplete, this can lead to expression imbalance and sex-biased gene expression. Recent work has uncovered an intriguing and variable pattern of dosage compensation across species that includes a lack of complete dosage compensation in ZW species compared with XY species. This has led to the hypothesis that ZW species do not require complete compensation or that complete compensation would negatively affect their fitness. To date, only one study, a study of the moth *Bombyx mori,* has discovered evidence for complete dosage compensation in a ZW species. We examined another moth species, *Manduca sexta*, using high-throughput sequencing to survey gene expression in the head tissue of males and females. We found dosage compensation to be complete in *M. sexta* with average expression between the Z chromosome in males and females being equal. When genes expressed at very low levels are removed by filtering, we found that average autosome expression was highly similar to average Z expression, suggesting that the majority of genes in *M. sexta* are completely dosage compensated. Further, this compensation was accompanied by sex-specific gene expression associated with important sexually dimorphic traits. We suggest that complete dosage compensation in ZW species might be more common than previously appreciated and linked to additional selective processes, such as sexual selection. More ZW and lepidopteran species should now be examined in a phylogenetic framework, to understand the evolution of dosage compensation.

## Introduction

Sex chromosome dosage compensation is a genetic regulatory mechanism that equalizes the expression of the homogametic sex chromosome and autosomal genes in the heterogametic sex of diploid organisms with a chromosomal-based sex determination system (Ohno 1967; Mank et al. 2011). In the heterogametic sex (males in the XY system and females in the ZW system), sex-linked genes on the homogametic sex chromosome (X or Z) are present as single copies compared with two copies in the homogametic sex (XX or ZZ). In many animal species, this has resulted in the evolution of complete compensation, a global mechanism of expression regulation applied to entire sex chromosomes to balance the expression of X/Z and autosomal genes, and which leads to the equalization of homogametic sex chromosome expression between the sexes. For example, in some mammals (including humans), gene dose is equalized through the inactivation of one X chromosome in homogametic females (XX) during early development, coupled with an up-regulation of X-linked genes, or a down-regulation of autosomal genes that are functionally linked to X-linked genes (Lyon 1961, 1999; Nguyen and Disteche 2006; Lin et al. 2007; Johnston et al. 2008; Julien et al. 2012). This equalizing of expression level leads to balanced transcription rates between males and females and preserves the integrity of gene networks that include both autosomal and sex-linked genes. However, not all mechanisms of dosage compensation are sex chromosome wide, and some species appear to compensate only a subset of genes on the X or Z (incomplete dosage compensation; Mank 2013). Emerging evidence suggests that patterns of dosage compensation are highly variable across sex-determination system and species (Mank et al. 2011).

The evolution of sex chromosomes proceeds with the differentiation of initially homologous chromosomes that accumulate antagonistic mutations, leading to selection for reduced recombination in the heterogametic sex and the gradual degradation of one homolog (Y and W in the XY and ZW systems, respectively; Bull 1983; Rice 1987; Charlesworth 1991; Charlesworth B and Charlesworth D 2000). Dosage compensation is thought to have evolved to compensate for this degradation (Ohno 1967). There is increasing evidence for a spectrum in the extent of sex chromosome dosage compensation across species ranging from complete to incomplete compensation, applied through global and gene-by-gene mechanisms of gene expression regulation (Vicoso and Bachtrog 2009). For example, although humans display complete dosage compensation, recent evidence suggests that approximately 15% of genes escape compensation on the X chromosome (Carrel and Willard 2005). Egg laying monotreme mammals (platypus and echidna, XY species) show a gene-by-gene pattern of incomplete dosage compensation of the X chromosome, whereas marsupials demonstrate X-inactivation and up-regulation of X expression through gene-by-gene effects that vary between tissues and species (McKay et al. 1987; Cooper et al. 1993; Deakin et al. 2009; Julien et al. 2012). Dosage compensation in *Drosophila* (XY) species is complete and achieved through the doubling of X chromosome expression in males (Mukherjee and Beermann 1965; Belote and Lucchesi 1980; Straub et al. 2005). In contrast, the ZW trematode parasite, *Schistosoma mansoni*, has no global mechanism of chromosomal dosage compensation (Vicoso and Bachtrog 2011), demonstrating gene-by-gene effects. ZW chickens and zebra finches also lack a global mechanism of dosage compensation (Mank and Ellegren 2009; Zhang et al. 2010; Ayers et al. 2013), yet their patterns of gene-by-gene effects differ (Itoh et al. 2010). Further, the flour beetle, *Tribolium castaneum* (XY), is thought to exhibit global X compensation to balance X and autosomal expression, yet female X expression is also upregulated leading to sex-biased gene expression (Prince et al. 2010). The variability in the degree of dosage compensation across species with differentiated sex chromosomes suggests that dosage compensation may not be a requirement for sex chromosome evolution and that other selective forces may shape dosage compensation evolution (Mank et al. 2011; Lin et al. 2012). Additionally, the complex phylogenetic distribution of dosage compensation indicates that it has evolved independently a number of times and highlights the importance of gene dose regulation to organismal fitness (Straub and Becker 2007; Julien et al. 2012; Livernois et al. 2013).

Incomplete dosage compensation produces an expression imbalance between males and females and is likely to be responsible for a greater proportion of sex-biased expression of Z/X-linked genes than previously assumed (Parsch and Ellegren 2013). Dosage compensation evolved during the evolution of sex chromosomes. Thus, the evolution of sex chromosomes, dosage compensation, and sex-biased gene expression are likely to be constrained by the selective processes acting on each. Several factors might influence the evolution of dosage compensation and sex-biased gene expression, such as the concentration of genes on a chromosome that require dosage compensation and the strength of divergent selection between the sexes (Livernois et al. 2012). It is unclear how often sex-biased gene expression is due to incomplete dosage compensation, and although sex is determined by the sex chromosomes, sex-specific traits are not necessarily controlled by sex chromosome genes (Mank 2009). However, the enrichment of sex-biased genes on the homogametic sex chromosome is seen frequently in ZW species where dosage compensation is rarely complete (Kaiser and Ellegren 2006; Arunkumar et al. 2009; Zhang et al. 2010).

The majority of ZW species demonstrate a pattern of incomplete dosage compensation, suggesting that complete compensation might be limited to male heterogametic species (Mank 2013). Numerous studies, focusing mainly on birds but also including snakes and Lepidoptera, have found incomplete dosage compensation across ZW species (Mank and Ellegren 2009; Itoh et al. 2010; Naurin et al. 2012; Adolfsson and Ellegren 2013; Vicoso, Emerson, et al. 2013), leading to the conclusion that such mechanisms may not exist in the ZW system (Wolf and Bryk 2011). This suggests that heterogametic females might be less sensitive to gene dose or that the presence of male-adapted genes on the Z, due to higher Z mutation rates and effective population sizes in males (dependent on several demographic and biological factors, see Mank et al. 2010), requires mitigation of antagonistic effects through lower female expression (Naurin et al. 2010). This means that incomplete dosage compensation might be a common cause of sex-biased gene expression in the ZW system. It should be noted that incomplete compensation leading to homogametic-biased expression is not unique to the ZW system. For example, the stickleback, *Gasterosteus aculeatus* (XY), has no chromosome-wide method of compensation and a higher number of female-biased genes on the X chromosome (Leder et al. 2010). Lepidoptera are female heterogametic, and the Indian meal moth, *Plodia interpunctella*, lacks complete Z chromosome dosage compensation (Harrison et al. 2012). Incomplete dosage compensation was also discovered in the silkworm *Bombyx mori* using microarray data (Zha et al. 2009). However, that result was recently challenged when complete Z dosage compensation was shown between males and females using high-throughput mRNA sequencing (Walters and Hardcastle 2011). The latter study is the only example of complete and globally applied dosage compensation of a Z chromosome. Walters and Hardcastle (2011) also saw that autosomal expression was elevated compared with Z-linked expression, an unexpected result under the expectation of complete compensation. Previous microarray-based studies of dosage compensation have been called into question (e.g., Xiong et al. 2010), and recent work has examined how different methodological aspects of evaluating gene expression can affect the conclusions drawn from dosage compensation studies (Jue et al. 2013). It is becoming clear that best practices need to be developed to examine the evolution of dosage compensation in a comparative manner (Mank 2013).

Here, we assess dosage compensation and sex-biased gene expression using high-throughput sequencing of mRNA from the heads of adult males and females of *Manduca sexta*, a moth species without a fully sequenced genome. We find that average gene expression levels between males and females, and between the Z chromosome and autosomes, were equal, providing evidence for a global mechanism of complete dosage compensation along the *M. sexta* Z chromosome. This result highlights the substantial variability in presence and degree of dosage compensation in the ZW system in general, and Lepidoptera in particular. Further, we found that 1,385 contigs were significantly differentially expressed (DE) between males and females, with two thirds of these genes being female biased. These sex-biased genes were distributed throughout the *M. sexta* genome, with no overrepresentation on the Z chromosome, confirming a lack of male-biased Z chromosome expression in *M. sexta* that is normally associated with incomplete dosage compensation in ZW species. A functional analysis of sex-biased genes demonstrated significant enrichment for a number of sex-related functions, including alternatively spliced genes, adult behavior, developmental processes, and, interestingly, sensory perception of light and neurological development relating to olfaction.

## Materials and Methods

### Sequencing and Assembly of the *M*. *sexta* Transcriptome

*Manduca sexta* larvae were raised on a wheat germ-based artificial diet in a climate-controlled chamber at 26 °C, light 16 h/25 °C dark 8 h cycle. To synchronize the eclosion time of the adults, the pupae were kept in a climate-controlled chamber with the following cycles: 14 °C, light 16 h/14 °C dark 8 h cycle for 3 weeks, and then at 27 °C, light 12 h/25 °C dark 12 h cycle for 2.5–3 weeks. All pupae were reared separately, based on gender, and four replicate samples were collected for each sex. Adult heads were collected between 15 and 20 h after eclosion, then immediately stored in RNAlater at −20 °C for RNA extraction. RNA was isolated using an RNeasy Mini Kit (Qiagen), following the manufacturer’s protocol. The RNA-seq libraries were constructed using a strand-specific RNA-Seq method according to Zhong et al. (2011). Briefly, polyA-RNA was isolated using Dynabeads Oligo(dT)25 (Invitrogen), then simultaneously eluted and fragmented in SuperScript III buffer. First-strand cDNA was synthesized using SuperScript III (Invitrogen). Second-strand cDNA was synthesized using RNase H (NEB) and DNA polymerase I (NEB) with a dUTP mix. The double-stranded cDNA fragments were then sequentially subjected to end repair, dA tailing, Y-shape adapter ligation, and Uracil DNA Glycosylase (NEB) treatment. Finally, the product was purified and polymerase chain reaction (PCR) amplified with indexed PCR primers. Sequencing (50 bp single-end reads) was performed on the Illumina HiSeq2000 platform at Weill Cornell Medical College.

The eight RNA-seq samples were used to perform a de novo transcriptome assembly using the Trinity pipeline (Grabherr et al. 2011; Haas et al. 2013). Trinity assembles sequenced reads based on sequence similarity using de Brujin graphs, constructing reads into contigs that represent genes and alternatively spliced transcripts. Once assembled, each library was separately mapped back to the assembly using RSEM (Li and Dewey 2011), and count levels of reads within each genomic feature (contigs) were extracted. Illumina reads for each library are available as fastq files in the ArrayExpress database (www.ebi.ac.uk/arrayexpress, last accessed March 6, 2014) under accession number E-MTAB-2066. The FASTA file containing contigs from the Trinity assembly was deposited in Dryad under data identifier doi:10.5061/dryad.gb135.

### Physical Locations of Genes and Dosage Compensation

To determine the physical positions of *M. sexta* genes, de novo assembled contigs were compared with the annotated genome of the silkworm, *B. mori*, using the Basic Local Alignment Search Tool (Blast; Altschul et al. 1990). There is a high level of synteny between the Z chromosomes of butterfly and moth species thus far sequenced (*Bombyx*, Mita et al. 2004; Xia et al. 2004; *Danaus*, Zhan et al. 2011; *Heliconius*, Dasmahapatra et al. 2012), making it possible to assign the putative physical locations of *M. sexta* Z and autosomal contigs through orthology to *B. mori* (Harrison et al. 2012). A reciprocal best-hit approach was used to determine highly similar 1:1 orthologs using a strict *e*-value cut off of 1 × 10^−10^. *Manduca sexta* contigs were assigned putative chromosome locations from their corresponding 1:1 orthologs in *B. mori*. All contigs analyzed were more than 200 bp in length, and fragments per kilobase per million mapped reads (FPKM), a conversion factor that accounts for contig length when considering expression levels, was calculated for each contig. Libraries were normalized using the trimmed mean of *M* values (TMM) normalization method in the NOISeq R package (Tarazona et al. 2011) before assessing dosage compensation. The four replicate male and female libraries were tested for uniformity using a Spearman's rank order correlation test. FPKM values for same-sex replicate libraries were highly correlated (pairwise Spearman's ρ > 0.93; *P < *2.2 × 10^−16^ between female replicates, and ρ > 0.91; *P < *2.2 × 10^−16^ between males), thus male and female replicates could be reliably combined to compare expression levels across Z and autosomal contigs. From the Blast results, contigs were partitioned into test sets for putative *M. sexta* autosomal and Z chromosome genes. Male:female gene expression ratio was investigated by log_2_ transforming FPKM for all contigs in the test set, as well as for genes with 1:1 orthologs in *B. mori*, to ensure there was no bias caused by removing contigs with reduced orthology.

Analyses of chromosome-wide expression data have been criticized for the potential bias introduced by a lack of robust filtering (Xiong et al. 2010; Kharchenko et al. 2011). Filtering of microarray data is common due to levels of background noise, below which gene expression cannot be accurately called. RNA sequencing can also be biased, depending on factors such as sequencing depth, and can influence conclusions as to the extent of dosage compensation (Kharchenko et al. 2011; Walters and Hardcastle 2011; Jue et al. 2013). Therefore, we explored our data using two different filtering methods used in previous investigations of dosage compensation, as well as assessing the unfiltered data set. First, filtering was performed as per Jue et al. (2013) to remove outlier contigs from the distribution of expression levels. The FPKM of each contig was log_2_ transformed, and outliers were removed if they were either 1) more than 1.5 times the mid-50 percentile above the 75^th^ percentile or 2) the same distance below the 25^th^ percentile, thus producing data that approximates a normal distribution. Second, our test set of contigs was filtered (using unlogged FPKM values) to remove low-expressed genes as per Harrison et al. (2012). We explored three separate data sets with different stringencies of this filtering method, discarding contigs that had expression levels below 2, 3, and 4 FPKM in at least four groups. Using these differing filtering methods for contigs with putative physical locations, we compared chromosome-wide expression patterns of the Z and autosomes, between males and females. Significance testing of distributions of FPKM values was performed using a two-sample Wilcoxon rank sum test, and for normally distributed log_2_ transformed data, *t*-tests were employed.

### Gene Expression Analysis and Functional Enrichment

Raw count values from read mapping to the de novo assembly were extracted using Trinity perl scripts for each of the four male and four female libraries. The edgeR package in R was used to perform differential expression analysis to identify DE contigs between males and females (Robinson et al. 2010). Normalization was performed in edgeR, using the TMM method and included adjustments for library size to account for between-sample variation in sequencing depth. For data sets with replicate samples, edgeR performs a generalized linear model using a negative binomial distribution to identify DE genes. Significantly, DE contigs were determined by using the false discovery rate (FDR) of Storey and Tibshirani (2003) to adjust gene-specific *P* values for multiple tests, taking a significance threshold of 5% FDR. A heatmap was constructed using the normalized counts of significant contigs to plot changes in moderated log_2_ counts per million (log CPM) across libraries, with the R package Heatplus (Ploner 2012). To address the possibility that edgeR does not sufficiently normalize libraries that have unequal sequencing depth, we performed differential analysis on a subsampled data set. Briefly, we down-sampled sequence libraries to the smallest library size, thereby equalizing all library sizes. Down-sampled libraries were mapped to the assembled contigs and differential expression analysis was repeated, as for the full data set.

To examine the role of coexpressed genes, functional enrichment tests can provide a statistically based summary of functional terms that occur more often than chance in a gene test set. Functional enrichment was performed using orthologous genes in *Drosophila melanogaster*, obtained through a Blast comparison of all *M. sexta* contigs to the *D. melanogaster* genome (assembly BDGP5, release 71). A Blast hit was considered orthologous when the best top hit passed an *e-*value threshold of less than 1 × 10^−5^. Functional enrichment of these orthologs was performed in GOrilla (Eden et al. 2009) and DAVID v6.7 (Dennis et al. 2003; Huang et al. 2009), testing the functions of *Drosophila* orthologs of significant *M. sexta* sex-biased contigs against the *D. melanogaster* gene set. The GOrilla functional enrichment test produces a list of significant individual gene ontology (GO) terms, whereas DAVID performs enrichment tests using a number of different functional terms, including GO terms, SwissProt keywords, and UniProt sequence features. Additionally, DAVID performs a fuzzy heuristic partitioning that groups GO terms by their degree of overlapping gene sets, reducing the presence of repetitive or nested terms. GOrilla GO terms and DAVID clusters were considered significant with an FDR < 5% and with an enrichment score (the geometric mean of annotation *P* values) of more than 1.3, respectively. DAVID clusters were filtered by hand to remove obvious overlapping clusters.

## Results

### Dosage Compensation

The *M. sexta* de novo transcriptome assembly resulted in a total of 31,459 gene contigs, with a total combined length of 23.9 Mb and an average per nucleotide transcriptome coverage of 34.7× for uniquely mapped reads across eight libraries, which ranged in size from 16.6 to 24.3 million reads. To assess the presence or absence of dosage compensation in *M. sexta,* assembled contigs were Blast searched against the *B. mori* genome to obtain the physical locations of 1:1 orthologs. We found 575 putative Z chromosome and 13,799 autosomal *M. sexta* contigs that had 1:1 orthology to *B. mori* genes and were more than 200 bp length. Filtering was performed to reduce bias from extreme expression levels, using two different methods. The number of retained putative *M. sexta* Z contigs under these different methods ranged from 451 to 568, and 11,956 to 13,689 for autosomal contigs (table 1), depending on the stringency of filtering. All pairwise correlations of FPKM values between replicate samples were highly significant (*P* < 2.2 × 10^−16^), and thus, averaged expression levels could be assessed for dosage compensation.

**Table 1:** Effect of Filtering Method and Stringency on the Number of Genes in Each Analysis and the Presence of Dosage Compensation

Filtering Method	Number of Z Genes Retained	Number of Autosomal Genes Retained	Z, M:F Parity*	M, Z:A Parity**	F, Z:A Parity***
Unfiltered	575	13,799	0.81	5.78 × 10^−05^	1.52 × 10^−06^
outlier removal	568	13,689	0.80	2.26 × 10^−04^	3.06 × 10^−05^
Remove genes < 2 FPKM	559	13,551	0.83	2.02 × 10^−04^	6.26 × 10^−06^
Remove genes < 3 FPKM	503	12,874	0.93	0.05	0.01
Remove genes < 4 FPKM	451	11,956	0.96	0.96	0.12

Note.—Filtering methods are outlined in the Materials and Methods section, genes were assigned location using 1:1 orthologs in *Bombyx mori*. Sex chromosome dosage compensation was assessed by comparing Z chromosome expression between males and females, and Z expression was compared with autosome expression for males and females separately. All significance tests were Wilcoxon tests except for the normally distributed outlier method of filtering, where a *t*-test was employed. Significance indicates disparity in expression levels.

*P values for Z, M:F parity compare average Z chromosome expression between males and females.

**P values for M, Z:A parity compare average male expression between Z and autosomal genes.

***P values for F, Z:A parity compare average female expression between Z and autosomal genes.

**Fig. 1.—:**
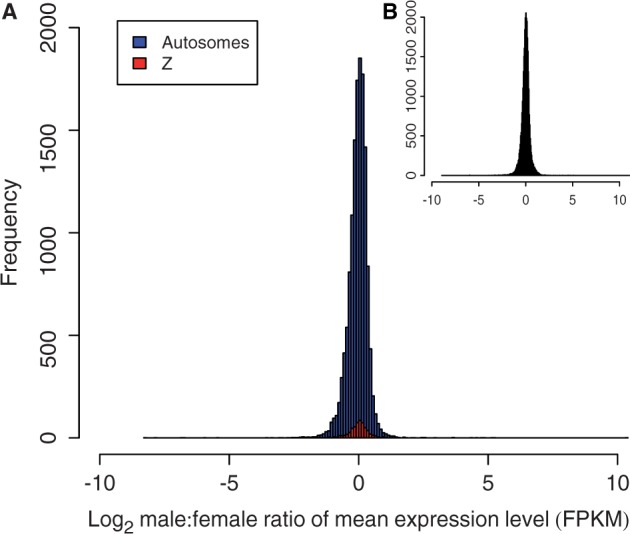
Histogram showing log_2_ male:female ratio of expression level (FPKM) for *Manduca sexta* contigs located on the Z chromosome (red) and the autosomes (blue; *a*), and all contigs regardless of physical location (*b*).

**Fig. 2.—:**
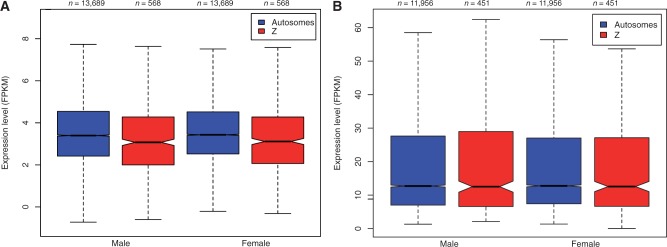
Average Z-linked and autosomal contig expression levels (FPKM) across four replicate males and females using the log_2_ outlier removal method of filtering (*a*) and removal of contigs with less than 4 FPKM (*b*). Black lines are the median of the FPKM distribution across contigs, boxes show the interquartile range, whiskers extend to 1.5 × the interquartile range and notches approximate the 95% confidence intervals of the medians. Overlapping notches are evidence for the similarity of median values.

In general, comparisons of global gene expression between males and females revealed minimal differences, and this was true for both the Z chromosome and for autosomes. Thus, these observations indicate complete dosage compensation in *M. sexta* (figs. 1 and 2). The distributions of male:female gene expression ratios between contigs on the Z chromosome and the autosomes overlapped completely (fig. 1*a*). Considering all assembled contigs together (i.e., regardless of physical location) resulted in a unimodal distribution (fig. 1*b*), indicating male:female parity. Autosomal expression differences between males and females were small (supplementary table S1, Supplementary Material online; fig. 2), matching the expectation of autosomal parity between males and females. However, the average expression level of autosome compared with Z chromosome contigs did show a small but variable difference that was dependent on filtering stringency (table 1).

Average Z chromosome expression between males and females was nearly identical and did not differ significantly at any level of filtering (Wilcoxon test; *P* > 0.05 for all comparisons; table 1, supplementary table S1, Supplementary Material online), indicating that the presence or level of filtering stringency did not affect the conclusion that the Z chromosome shows complete dosage compensation in *M. sexta*. However, a significant, though small, difference was seen between average autosome and Z expression in both males and females when data were unfiltered or filtered at low stringency (unfiltered, log_2_ transformed outlier removal and data filtered at 2 FPKM; fig. 2*a*; table 1; supplementary fig. S1 and S2, Supplementary Material online). This pattern of increased autosomal expression was strongest in the unfiltered data set (Wilcoxon tests; female *P* = 1.52 × 10^−6^; male *P* = 5.78 × 10^−5^), but average expression differences and strength of significance reduced rapidly with a small increase in filtering threshold: Although differences were significant when filtering at 2 FPKM (female *P* = 6.26 × 10^−6^; male *P* = 2.02 × 10^−4^), they were only marginally significant at 3 FPKM (female *P* = 0.01; male *P* = 0.05) and nonsignificant when filtering at 4 FPKM (female *P* = 0.12; male *P* = 0.96; fig. 2*b*; table 1; supplementary figs. S1–S3, Supplementary Material online). Filtering at the 4 FPKM threshold resulted in removal of 21.6% of Z chromosome contigs and 13.4% of autosomal contigs (supplementary table S2, Supplementary Material online) and led to Z:autosome parity. Thus, our analysis reveals that when data from genes expressed at very low levels are removed by filtering, the remaining majority are fully dosage compensated and equal between the sexes. The outlier removal filtering method produced a test set of 568 Z chromosome contigs and 13,689 autosome contigs for comparison, which had a mean log_2_ FPKM of 3.65, 3.35, 3.6, and 3.33 for autosome female, Z female, autosome male, and Z male, respectively (fig. 2*a*, supplementary table S1, Supplementary Material online). Median expression levels when filtering at a threshold of 4 FPKM were 12.75, 12.55, 12.7, and 12.52 for autosome female, Z female, autosome male, and Z male, respectively (fig. 2*b*; supplementary table S1, Supplementary Material online).

### Differential Expression and Functional Analyses

**Fig. 3.—:**
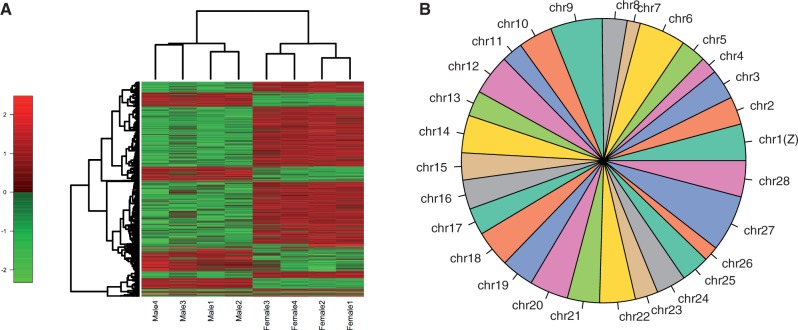
Heatmap of significantly DE genes (FDR < 0.05) plotted using log_2_ counts per million (log CPM) per gene for each *Manduca sexta* library (males 1–4 and females 1–4, as labeled; *a*). Color bar legend indicates the degree of log CPM change between males and females. (*b*) Physical locations of sex-biased genes determined using 1:1 orthologs in *Bombyx mori*. Chromosome numbers are shown, with the Z chromosome being chromosome 1, as indicated. The size of each chromosome segment in the pie chart represents the relative abundance of sex-biased genes, normalized to chromosome size.

Through differential expression analysis of males and females, we identified a total of 1,385 sex-biased contigs from the full data set (FDR < 5%). The majority of these sex-biased contigs (960) were upregulated in females (female-biased; fig. 3*a*). Because unequal library sizes may influence differential expression results using edgeR, we also performed differential expression on down-sampled libraries, which were of equal size, to compare to the full data set results. Analysis of this subsampled data demonstrated very similar results to that of the full data set. In total, 1,310 genes were significantly DE (FDR < 0.05), with 906 being female biased and 404 male biased, overlapping with the full data set results considerably (supplementary fig. S4, Supplementary Material online). Mapping efficiencies of the subsampled data were high and equal between libraries (>91% for all libraries; supplementary table S3, Supplementary Material online). Therefore, inefficient mapping and unequal library sizes are unlikely to have contributed to our full data set differential expression results. Thus, the full data set results were used for all further analyses.

To examine the distribution of sex-biased genes across the genome, DE contigs from the full data set (1,385 contigs) were assigned a putative physical location when they had 1:1 orthology to *B. mori*. Of the 1,385 sex-biased contigs, 604 had 1:1 orthologs with positional information. The physical locations of sex-biased contigs did not exhibit a bias toward the Z chromosome, as would be expected in the absence of dosage compensation, and sex-biased genes were distributed throughout the genome (fig. 3*b*). Average expression levels of sex-biased contigs between males and females from the Z chromosome and autosomes were also examined (570 autosomal contigs and 34 Z-linked contigs; supplementary fig. S5, Supplementary Material online). The average expression of sex-biased contigs from the Z chromosome appeared higher than that of contigs from the autosomes; however, this effect was not significant (Wilcoxon test; *P* > 0.05).

Functional enrichment of sex-biased contigs was performed using *D. melanogaster* orthologs obtained from a Blast search of *D. melanogaster* cDNAs. In total, 5,793 contigs had good hits to genes in the *D. melanogaster* genome, representing 18.4% of *M. sexta* contigs. Of these 5,793 contigs, 191 were sex biased in the differential expression analysis, and thus, functional information from their *D. melanogaster* orthologs could be used to perform functional enrichment tests. Generally, both DAVID and GOrilla demonstrated significant enrichment of similar GO terms, with DAVID producing a more succinct set of functional clusters (table 2).

**Table 2:** DAVID Functional Enrichment Results

	Annotation Category	Annotation Term	Gene Count	*P*	FDR
Annotation cluster 1	SP_PIR_KEYWORDS	Alternative splicing	34	1.77 × 10^−13^	3.11 × 10^−11^
Enrichment score: 9.11	UP_SEQ_FEATURE	Splice variant	34	3.10 × 10^−08^	9.90 × 10^−06^
	SP_PIR_KEYWORDS	Phosphoprotein	30	8.35 × 10^−08^	4.90 × 10^−06^
Annotation cluster 2	GOTERM_BP_FAT	GO:0016056 rhodopsin-mediated signaling pathway	6	4.57 × 10^−06^	1.23 × 10^−03^
Enrichment score: 3.09	GOTERM_BP_FAT	GO:0007602 phototransduction	8	5.31 × 10^−06^	1.15 × 10^−03^
	GOTERM_BP_FAT	GO:0050953 sensory perception of light stimulus	9	1.32 × 10^−05^	2.04 × 10^−03^
Annotation cluster 3	GOTERM_CC_FAT	GO:0044459 plasma membrane part	25	3.02 × 10^−08^	5.44 × 10^−06^
Enrichment score: 2.81	GOTERM_CC_FAT	GO:0005886 plasma membrane	28	1.46 × 10^−04^	8.71 × 10^−03^
	GOTERM_CC_FAT	GO:0005887 integral to plasma membrane	11	1.86 × 10^−03^	4.09 × 10^−02^
Annotation cluster 4	GOTERM_BP_FAT	GO:0030001 metal ion transport	11	2.44 × 10^−04^	1.38 × 10^−02^
Enrichment score: 2.71	SP_PIR_KEYWORDS	Calcium transport	4	6.71 × 10^−04^	9.80 × 10^−03^
	GOTERM_BP_FAT	GO:0006816 calcium ion transport	5	1.14 × 10^−03^	4.79 × 10^−02^
Annotation cluster 5	SP_PIR_KEYWORDS	RNA editing	9	5.87 × 10^−09^	5.16 × 10^−07^
Enrichment score: 2.48	SP_PIR_KEYWORDS	Ion transport	12	1.08 × 10^−05^	3.78 × 10^−04^
	SP_PIR_KEYWORDS	Membrane	30	6.35 × 10^−05^	1.86 × 10^−03^
Annotation cluster 6	GOTERM_BP_FAT	GO:0009063 cellular amino acid catabolic process	5	1.64 × 10^−03^	6.12 × 10^−02^
Enrichment score: 2.28	GOTERM_BP_FAT	GO:0009069 serine family amino acid metabolic process	4	1.80 × 10^−03^	6.48 × 10^−02^
	GOTERM_BP_FAT	GO:0009310 amine catabolic process	5	2.27 × 10^−03^	7.86 × 10^−02^
Annotation cluster 7	GOTERM_BP_FAT	GO:0060284 regulation of cell development	11	3.52 × 10^−05^	3.45 × 10^−03^
Enrichment score: 2.13	GOTERM_BP_FAT	GO:0051960 regulation of nervous system development	8	4.90 × 10^−04^	2.38 × 10^−02^
	GOTERM_BP_FAT	GO:0045664 regulation of neuron differentiation	6	1.47 × 10^−03^	5.94 × 10^−02^
Annotation cluster 8	GOTERM_BP_FAT	GO:0007610 behavior	17	9.93 × 10^−04^	4.56 × 10^−02^
Enrichment score: 1.97	GOTERM_BP_FAT	GO:0007626 locomotory behavior	9	7.29 × 10^−03^	1.68 × 10^−01^
	GOTERM_BP_FAT	GO:0008344 adult locomotory behavior	6	9.92 × 10^−03^	2.05 × 10^−01^
Annotation cluster 9	GOTERM_BP_FAT	GO:0042659 regulation of cell fate specification	4	3.49 × 10^−03^	1.02 × 10^−01^
Enrichment score: 1.93	GOTERM_BP_FAT	GO:0010453 regulation of cell fate commitment	4	3.49 × 10^−03^	1.02 × 10^−01^
	GOTERM_BP_FAT	GO:0009996 negative regulation of cell fate specification	3	2.52 × 10^−02^	3.41 × 10^−01^
Annotation cluster 11	GOTERM_MF_FAT	GO:0008017 microtubule binding	6	3.51 × 10^−03^	9.25 × 10^−02^
Enrichment score: 1.76	GOTERM_MF_FAT	GO:0015631 tubulin binding	6	4.88 × 10^−03^	1.04 × 10^−01^
	INTERPRO	IPR002017 spectrin repeat	3	7.42 × 10^−03^	4.51 × 10^−01^
Annotation cluster 12	GOTERM_BP_FAT	GO:0046394 carboxylic acid biosynthetic process	5	1.75 × 10^−02^	2.85 × 10^−01^
Enrichment score: 1.64	GOTERM_BP_FAT	GO:0016053 organic acid biosynthetic process	5	1.75 × 10^−02^	2.85 × 10^−01^
	GOTERM_BP_FAT	GO:0008610 lipid biosynthetic process	6	3.83 × 10^−02^	4.02 × 10^−01^
Annotation cluster 16	GOTERM_BP_FAT	GO:0016319 mushroom body development	5	7.51 × 10^−03^	1.69 × 10^−01^
Enrichment score: 1.42	GOTERM_BP_FAT	GO:0048666 neuron development	13	7.84 × 10^−03^	1.72 × 10^−01^
	GOTERM_BP_FAT	GO:0030182 neuron differentiation	14	1.12 × 10^−02^	2.16 × 10^−01^
Annotation cluster 17	GOTERM_BP_FAT	GO:0051653 spindle localization	4	1.48 × 10^−03^	5.74 × 10^−02^
Enrichment score: 1.41	GOTERM_BP_FAT	GO:0051293 establishment of spindle localization	4	1.48 × 10^−03^	5.74 × 10^−02^
	GOTERM_CC_FAT	GO:0005938 cell cortex	6	8.83 × 10^−03^	1.01 × 10^−01^
Annotation cluster 18	GOTERM_BP_FAT	GO:0055080 cation homeostasis	4	1.44 × 10^−02^	2.48 × 10^−01^
Enrichment score: 1.38	GOTERM_BP_FAT	GO:0048878 chemical homeostasis	5	1.94 × 10^−02^	3.06 × 10^−01^
	GOTERM_BP_FAT	GO:0050801 ion homeostasis	4	4.29 × 10^−02^	4.16 × 10^−01^

Note.—All GO term clusters presented have an enrichment score of more than 1.3 (enrichment scores are shown underneath the cluster number). Enrichment scores are the geometric mean of GO term *P* values within a cluster. Each cluster is presented as the top three representative GO terms for that cluster and includes the number of sex-biased genes, annotated with each functional GO term, the *P* value and FDR. Annotation term notation: GOTERM, GO term; BP, biological process; CC, cellular component; MF, molecular function; FAT; DAVID database filtered for specific GO terms; SP_PIR_KEYWORDS, Swiss-Prot Protein Information Resource Keywords; UP_SEQ_FEATURE, UniProt sequence feature.

Nineteen DAVID clusters demonstrated an enrichment score of more than 1.3, 14 of which had nonredundant functions (table 2). Many of these GO terms were developmental (e.g., GO:0060284, regulation of cell development); however, several involved the sensing and parsing of external cues and changes in behavior. These included GO terms for the response to an external or abiotic stimulus that were specifically involved in sensory perception of light stimulus (GO:0050953; e.g., *ninaC*, *inaD*, and *trpl*). Behavior-related GO terms included behavior itself (GO:0007610; e.g., *cpo*, *b*, and *Syn*) and adult locomotory behavior (GO:0008344; e.g., *tutl*, *cac*, and *t*). Other clusters included annotations involved in the regulation of development, specifically regulation of nervous system development (GO:0051960; e.g., *CadN*, *shot*, and *sdk*), and neurological development that included the term mushroom body development (GO:0016319; e.g., *RhoGAPp190*, *stan*, and *chinmo*). The regulation of sex-specific gene expression was represented by two clusters, one containing annotations for alternative splicing and the other for RNA editing. GOrilla produced 102 significant GO terms in total (FDR < 5%; supplementary table S4, Supplementary Material online).

To visualize gene expression changes for contigs of a specific function, we examined the expression levels of four functional groups of contigs, chosen based on their inclusion in significant GO terms in the DAVID functional enrichment test. The four GO terms were phototransduction (8 genes; GO:0007602), deactivation of rhodopsin mediated signaling (5 genes; GO:0016059), regulation of cell development (11 genes; GO:0060284), and behavior (17 genes; GO:0007610). Mean expression levels (FPKM) of genes within each GO term were calculated across four replicates. Genes within each functional annotation were all upregulated in females compared with males, supporting the general pattern of female-biased gene expression (supplementary fig. S6 and table S5, Supplementary Material online).

## Discussion

Complete sex chromosome dosage compensation has not been unequivocally observed in a species with a ZW sex determination system, leading to the supposition that complete compensation of sex chromosome expression only occurs in male heterogametic species. In fact, only one study has shown complete dosage compensation in a ZW species, following a reassessment of previously published data (Zha et al. 2009; Walters and Hardcastle 2011). Our results show that complete dosage compensation has evolved in the ZW species *M. sexta*, alongside sex-specific gene expression. Regardless of the method and stringency of data filtering performed, the average expression of Z-linked contigs between males and females was essentially identical, suggesting the complete compensation of Z-linked genes. Further, in agreement with Walters and Hardcastle (2011), we found the average expression level of autosomal contigs to be higher than that of Z-linked contigs. However, this imbalance quickly disappears when contigs with low expression levels are removed (supplementary fig. S1–S3, Supplementary Material online, vs. fig. 2*b*), suggesting that the majority of genes in the *M. sexta* genome are fully dosage compensated between the Z chromosome and autosomes.

### Dosage Compensation

**Fig. 4.—:**
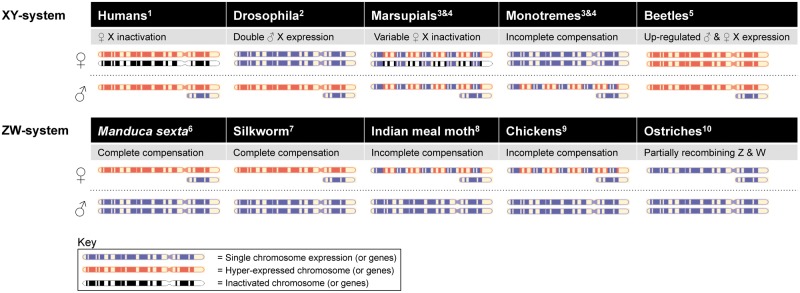
Putative models of X/Z chromosomal dosage compensation for representative XY and ZW species. Chromosomes represent the X/Z (large chromosomes) and Y/W (small chromosomes) across XY and ZW species, for males and females, and a brief summary of the putative mechanism is provided (if known; grey boxes). See Introduction for details. Blue chromosomes represent normal gene expression levels across chromosomes, red chromosomes represent upregulated gene expression, and black chromosomes inactivated chromosomal expression. References are as follows: ^1^Johnston et al. (2008); ^2^Straub et al. (2005); ^3^Deakin et al. (2009); ^4^Julien et al. (2012); ^5^Prince et al. (2010); ^6^Current study; ^7^Walters and Hardcastle (2011); ^8^Harrison et al. (2012); ^9^Itoh et al. (2010); ^10^Adolfsson and Ellegren (2013).

Dosage compensation has previously been considered as necessary for sex chromosome evolution, to equalize changes in gene product concentration as one chromosome degenerates. However, dosage compensation studies are often contradictory (Livernois et al. 2013), and patterns of dosage compensation are varied across species (fig. 4), ranging from incomplete compensation to the hyperexpression of sex chromosomes in both sexes (Prince et al. 2010). Incomplete dosage compensation exists in XY species, but is by far more common in the ZW system. It has been suggested that ZW species have not evolved complete compensation because either 1) heterogametic females are not highly adversely affected by unequal dose and require fewer genes on the Z to be compensated or 2) the presence of male-adapted genes on the Z has led to a trade-off between maintaining gene network integrity and expressing maladaptive genes in females (Mank and Ellegren 2009; Naurin et al. 2010; Mank et al. 2011; Naurin et al. 2012). However, we have discovered complete dosage compensation in a ZW species, alongside genome-wide sex-biased gene expression. This suggests that complete dosage compensation is required to balance the expression of key gene networks throughout the genome of *M. sexta*. The evolution of complete compensation may have allowed for the evolution of sex-biased expression through gene-by-gene mechanisms driven by sex-specific selective pressures such as sexual selection or sex-specific natural selection. Alternatively, sex-biased expression might have evolved alongside dosage compensation, under sexually antagonistic selection (Vicoso, Kaiser, et al. 2013). This might occur when specific genome-wide loci are under strong selection for optimal expression levels between the sexes, with dosage compensation of Z-linked genes allowing the fine tuning of sex-specific dose. The presence of complete compensation in a related species, *B. mori*, indicates that complete dosage compensation might have evolved in a common ancestor of these two species. Thus, further studies are necessary to determine whether complete compensation is common in moths, and in ZW species generally, and to improve our understanding of the phylogenetic distribution of compensation and the mechanisms that underlie it (fig. 4).

The resolution of conflict during sex chromosome evolution through gene-by-gene regulation of sex-linked genes is thought to be a counter mechanism to sex chromosome differentiation, allowing for sex determination with homomorphic sex chromosomes (e.g., in the Emu; Vicoso, Kaiser, et al. 2013) and thus not requiring the evolution of dosage compensation (e.g., Boa constrictor; Vicoso, Emerson, et al. 2013). This is an interesting aspect of the dynamics of sex chromosome evolution wherein both dosage compensation and sex-biased gene expression are essentially the result of sexual conflict over optimal trait values for males and females, yet the target loci of these processes may differ (Mank et al. 2011). Therefore, complete sex chromosome dosage compensation may not be required during sex chromosome evolution in ZW species but in some instances may have evolved alongside gene-by-gene mechanisms for key sex-specific loci across the genome. The signal of female-biased gene expression seen here is unusual for ZW species, which generally demonstrate expression of male-biased Z-linked genes due to incomplete dosage compensation of the Z chromosome (e.g., in birds; Naurin et al. 2012). Thus, female-biased gene expression is more common in XY species where dosage compensation is more frequently complete. It is likely that sex-biased gene expression is a result of sexual selection in *M. sexta*, as sex-biased genes were found to function in known sexually dimorphic traits (see later). This indicates that some patterns of sex-specific expression seen on the Z in other ZW species may also be due to sex-specific selection (Mank et al. 2011).

### Sex-Biased Genes in *M. sexta*

We found a number of contigs to be DE between the sexes, and enrichment analyses suggested that these differences were associated with a number of sexually dimorphic functions. *Manduca sexta* is sexually dimorphic in antennal lobe morphology, with sex-specific differences in size and position of glomeruli (olfactory centers of the insect antennal lobe; Rospars and Hildebrand 2000). This underlies a general dimorphism in olfactory system and sensory physiology in *M. sexta* (Shields and Hildebrand 2001). These differences correlate with our expression data from male and female heads, with significant enrichment of genes relating to the regulation of nervous system and mushroom body development. Mushroom bodies are the next step in olfaction pathways after the antennal lobe, and they translate olfactory sensory information into learned behavioral responses (Matsumoto and Hildebrand 1981; Daly and Smith 2000; Zars 2000; Caron et al. 2013). Sex-biased genes in *M. sexta* were functionally enriched for sensory perception and behavior, including five genes involved in the sensory perception of smell (GO:0007608; *rdgA*, *rdgB*, *eag*, *norpA*, and *Ir8A*). Interestingly, there was a strong signal for genes involved in visual perception, suggesting that vision might also be an important sexually dimorphic trait in this species. *Manduca sexta* is a crepuscular/nocturnal species and thus vision might be of particular importance to female-specific behavior in low light environments, for example, in the detection of suitable host plants for oviposition. Indeed, the majority of sex-biased gene functions were toward female expression, suggesting that females have evolved specific expression of genes involved in visual and olfactory sensory perception and adult behavior. Gene expression has been previously surveyed in the antennae of *M. sexta* adults using microarray data (Grosse-Wilde et al. 2011). Grosse-Wilde et al. (2011) discovered a female bias in the number of transcripts found in the antennal tissue with 729 transcripts present in the female compared with 348 in the male. Together these data suggest that sex-specific selection has acted to regulate genes across the genome related to sensory perception and behavior in *M. sexta*.

### Conclusion

We examined dosage compensation using two methods of filtering and several different stringencies for filtering contigs with low expression levels. Increased stringency in filtering led to increased parity of Z:autosome expression. Walters and Hardcastle (2011) also saw disparity between Z-linked and autosome expression; however, we have shown that this is only true for a minority of genes. Thus, filtering and analysis methods are important for a thorough investigation of the extent of dosage compensation. Analytical protocols including the initial experimental design, for example, the developmental stage of the organism and the tissue assayed, and the sequencing and mapping or assembly method can all influence conclusions on dosage compensation (Jue et al. 2013). Our result of complete dosage compensation between male and female Z-linked genes held true across all filtering methods and was robust to potential errors in determining physical locations (i.e., the unimodal distribution of all male:female contigs; fig. 1*b*). Further, complete chromosome and sex parity was true for at least three-quarters of the most highly expressed genes. The sampling of adult heads allowed us to survey the effect of dosage compensation after sex determination and assay sex-specific gene expression at an important life stage, which included the sexually dimorphic antennal tissue. Only a subset of orthologous sex-biased contigs was assessed for function, due to few Blast hits to *Drosophila* genes. Ascertaining gene functions for nonmodel organisms can often be a challenge when comparing genes with that of a divergent model species. Thus, although we recovered a number of sex-biased genes with functional annotations for known sexually dimorphic traits in *M. sexta*, it is likely that some important functions were missed. The improved annotation of lepidopteran genomes, and the increasing number of genomes being sequenced, will aid in the improvement of functional annotation of *M. sexta* genes.

The evolution of sex chromosomes, dosage compensation, sex-specific gene expression, and sex determination all involve the specific regulation of sex-related genes. Each process may be important at different developmental stages, and the expression of these genes as networks requires their tight regulation across the genome. We find that *M. sexta* fully compensates for Z chromosome dose between males and females and with autosomal expression. This indicates that *M. sexta* requires balance in genome-wide gene network expression, and this balance might be necessary for the sex-biased expression of autosomal genes that underlie important sexually dimorphic traits. We propose that sex-specific selection may be an important influence on the evolution of complete dosage compensation in ZW species and that additional ZW taxa should be examined to determine the extent of compensation and sex-biased expression in a phylogenetic framework.

## Supplementary Material

Supplementary figures S1–S6 and tables S1–S8 are available at *Genome Biology and Evolution* online (http://www.gbe.oxfordjournals.org/).

Supplementary Data

## References

[evu035-B1] Adolfsson S, Ellegren H (2013). Lack of dosage compensation accompanies the arrested stage of sex chromosome evolution in ostriches. Mol Biol Evol..

[evu035-B2] Altschul SF, Gish W, Miller W, Myers EW, Lipman DJ (1990). Basic local alignment search tool. J Mol Biol..

[evu035-B3] Arunkumar KP, Mita K, Nagaraju J (2009). The silkworm Z chromosome is enriched in testis-specific genes. Genetics.

[evu035-B4] Ayers KL (2013). RNA sequencing reveals sexually dimorphic gene expression before gonadal differentiation in chicken and allows comprehensive annotation of the W-chromosome. Genome Biol..

[evu035-B5] Belote JM, Lucchesi JC (1980). Control of X chromosome transcription by the *maleless* gene in *Drosophila*. Nature.

[evu035-B6] Bull JJ (1983). Evolution of sex determining mechanisms.

[evu035-B7] Caron SJC, Ruta V, Abbott LF, Axel R (2013). Random convergence of olfactory inputs in the *Drosophila* mushroom body. Nature.

[evu035-B8] Carrel L, Willard HF (2005). X-inactivation profile reveals extensive variability in X-linked gene expression in females. Nature.

[evu035-B9] Charlesworth B (1991). The evolution of sex-chromosomes. Science.

[evu035-B10] Charlesworth B, Charlesworth D (2000). The degeneration of Y chromosomes. Philos Trans R Soc Lond B Biol Sci..

[evu035-B11] Cooper DW, Johnston PG, Watson JM, Graves JAM (1993). X-inactivation in marsupials and monotremes. Semin Dev Biol..

[evu035-B12] Daly KC, Smith BH (2000). Associative olfactory learning in the moth *Manduca sexta*. J Exp Biol..

[evu035-B13] Dasmahapatra KK (2012). Butterfly genome reveals promiscuous exchange of mimicry adaptations among species. Nature.

[evu035-B14] Deakin JE, Chaumeil J, Hore TA, Graves JA (2009). Unravelling the evolutionary origins of X chromosome inactivation in mammals: insights from marsupials and monotremes. Chromosome Res..

[evu035-B15] Dennis JG (2003). DAVID: database for annotation, visualization, and integrated discovery. Genome Biol..

[evu035-B16] Eden E, Navon R, Steinfeld I, Lipson D, Yakhini Z (2009). GOrilla: a tool for discovery and visualization of enriched go terms in ranked gene lists. BMC Bioinformatics.

[evu035-B17] Grabherr MG (2011). Full-length transcriptome assembly from RNA-Seq data without a reference genome. Nat Biotechnol..

[evu035-B18] Grosse-Wilde E (2011). Antennal transcriptome of *Manduca sexta*. Proc Natl Acad Sci U S A..

[evu035-B19] Haas BJ (2013). *De novo* transcript sequence reconstruction from RNA-Seq using the trinity platform for reference generation and analysis. Nat Protoc..

[evu035-B20] Harrison PW, Mank JE, Wedell N (2012). Incomplete sex chromosome dosage compensation in the indian meal moth, *Plodia interpunctella,* based on de novo transcriptome assembly. Genome Biol Evol..

[evu035-B21] Huang DW, Sherman BT, Lempicki RA (2009). Systematic and integrative analysis of large gene lists using DAVID bioinformatics resources. Nat Protoc..

[evu035-B22] Itoh Y (2010). Sex bias and dosage compensation in the zebra finch versus chicken genomes: general and specialized patterns among birds. Genome Res..

[evu035-B23] Johnston CM (2008). Large-scale population study of human cell lines indicates that dosage compensation is virtually complete. PLoS Genet..

[evu035-B24] Jue NK (2013). Determination of dosage compensation of the mammalian X chromosome by RNA-Seq is dependent on analytical approach. BMC Genomics.

[evu035-B25] Julien P (2012). Mechanisms and evolutionary patterns of mammalian and avian dosage compensation. PLoS Biol..

[evu035-B26] Kaiser VB, Ellegren H (2006). Nonrandom distribution of genes with sex-biased expression in the chicken genome. Evolution.

[evu035-B27] Kharchenko PV, Xi RB, Park PJ (2011). Evidence for dosage compensation between the X chromosome and autosomes in mammals. Nat Genet..

[evu035-B28] Leder EH (2010). Female-biased expression on the X chromosome as a key step in sex chromosome evolution in threespine sticklebacks. Mol Biol Evol..

[evu035-B29] Li B, Dewey CN (2011). RSEM: accurate transcript quantification from RNA-Seq data with or without a reference genome. BMC Bioinformatics.

[evu035-B30] Lin F, Xing K, Zhang J, He X (2012). Expression reduction in mammalian X chromosome evolution refutes Ohno's hypothesis of dosage compensation. Proc Natl Acad Sci U S A..

[evu035-B31] Lin H (2007). Dosage compensation in the mouse balances up-regulation and silencing of X-linked genes. PLoS Biol..

[evu035-B32] Livernois AM, Graves JA, Waters PD (2012). The origin and evolution of vertebrate sex chromosomes and dosage compensation. Heredity.

[evu035-B33] Livernois AM (2013). Independent evolution of transcriptional inactivation on sex chromosomes in birds and mammals. PLoS Genet..

[evu035-B34] Lyon MF (1961). Gene action in the X-chromosome of the mouse (*Mus musculus* l.). Nature.

[evu035-B35] Lyon MF (1999). X-chromosome inactivation. Curr Biol..

[evu035-B36] Mank JE (2009). The w, x, y and z of sex-chromosome dosage compensation. Trends Genet..

[evu035-B37] Mank JE (2013). Sex chromosome dosage compensation: definitely not for everyone. Trends Genet..

[evu035-B38] Mank JE, Ellegren H (2009). All dosage compensation is local: gene-by-gene regulation of sex-biased expression on the chicken Z chromosome. Heredity.

[evu035-B39] Mank JE, Hosken DJ, Wedell N (2011). Some inconvenient truths about sex chromosome dosage compensation and the potential role of sexual conflict. Evolution.

[evu035-B40] Mank JE, Vicoso B, Berlin S, Charlesworth B (2010). Effective population size and the faster-X effect: empirical results and their interpretation. Evolution.

[evu035-B41] Matsumoto SG, Hildebrand JG (1981). Olfactory mechanisms in the moth *Manduca sexta:* response characteristics and morphology of central neurons in the antennal lobes. Proc Roy Soc Lond B Biol Sci..

[evu035-B42] McKay LM, Wrigley JM, Graves JA (1987). Evolution of mammalian X-chromosome inactivation: sex chromatin in monotremes and marsupials. Aust J Biol Sci..

[evu035-B43] Mita K (2004). The genome sequence of silkworm, *Bombyx mori*. DNA Res..

[evu035-B44] Mukherjee AS, Beermann W (1965). Synthesis of ribonucleic acid by the X-chromosomes of *Drosophila melanogaster* and the problem of dosage compensation. Nature.

[evu035-B45] Naurin S, Hansson B, Bensch S, Hassequist D (2010). Why does dosage compensation differ between XY and ZW taxa?. Trends Genet..

[evu035-B46] Naurin S, Hasselquist D, Bensch S, Hansson B (2012). Sex-biased gene expression on the avian Z chromosome: highly expressed genes show higher male-biased expression. PLoS One.

[evu035-B47] Nguyen DK, Disteche CM (2006). Dosage compensation of the active X chromosome in mammals. Nat Genet..

[evu035-B48] Ohno S (1967). Sex chromosomes and sex-linked genes.

[evu035-B49] Parsch J, Ellegren H (2013). The evolutionary causes and consequences of sex-biased gene expression. Nat Rev Genet..

[evu035-B50] Ploner A (2012). Heatplus: heatmaps with row and/or column covariates and colored clusters.

[evu035-B51] Prince EG, Kirkland D, Demuth JP (2010). Hyperexpression of the X chromosome in both sexes results in extensive female bias of X-linked genes in the flour beetle. Genome Biol Evol..

[evu035-B52] Rice WR (1987). The accumulation of sexually antagonistic genes as a selective agent promoting the evolution of reduced recombination between primitive sex-chromosomes. Evolution.

[evu035-B53] Robinson MD, McCarthy DJ, Smyth GK (2010). Edger: a bioconductor package for differential expression analysis of digital gene expression data. Bioinformatics.

[evu035-B54] Rospars JP, Hildebrand JG (2000). Sexually dimorphic and isomorphic glomeruli in the antennal lobes of the sphinx moth *Manduca sexta*. Chem Senses..

[evu035-B55] Shields VD, Hildebrand JG (2001). Recent advances in insect olfaction, specifically regarding the morphology and sensory physiology of antennal sensilla of the female sphinx moth *Manduca sexta*. Microsc Res Tech..

[evu035-B56] Storey JD, Tibshirani R (2003). Statistical significance for genomewide studies. Proc Natl Acad Sci U S A..

[evu035-B57] Straub T, Becker PB (2007). Dosage compensation: the beginning and end of generalization. Nat Rev Genet..

[evu035-B58] Straub T, Gilfillan GD, Maier VK, Becker PB (2005). The *Drosophila* MSL complex activates the transcription of target genes. Gene Dev..

[evu035-B59] Tarazona S, García-Alcalde F, Dopazo J, Ferrer A, Conesa A (2011). Differential expression in RNA-seq: a matter of depth. Genome Res..

[evu035-B60] Vicoso B, Bachtrog D (2009). Progress and prospects toward our understanding of the evolution of dosage compensation. Chromosome Res..

[evu035-B61] Vicoso B, Bachtrog D (2011). Lack of global dosage compensation in *Schistosoma mansoni*, a female-heterogametic parasite. Genome Biol Evol..

[evu035-B62] Vicoso B, Emerson JJ, Zektser Y, Mahajan S, Bachtrog D (2013). Comparative sex chromosome genomics in snakes: differentiation, evolutionary strata, and lack of global dosage compensation. PLoS Biol..

[evu035-B63] Vicoso B, Kaiser VB, Bachtrog D (2013). Sex-biased gene expression at homomorphic sex chromosomes in emus and its implication for sex chromosome evolution. Proc Natl Acad Sci U S A..

[evu035-B64] Walters JR, Hardcastle TJ (2011). Getting a full dose? Reconsidering sex chromosome dosage compensation in the silkworm, *Bombyx mori*. Genome Biol Evol..

[evu035-B65] Wolf JB, Bryk J (2011). General lack of global dosage compensation in ZZ/ZW systems? Broadening the perspective with RNA-Seq. BMC Genomics.

[evu035-B66] Xia QY (2004). A draft sequence for the genome of the domesticated silkworm (*Bombyx mori*). Science.

[evu035-B67] Xiong YY (2010). RNA sequencing shows no dosage compensation of the active X-chromosome. Nat Genet..

[evu035-B68] Zars T (2000). Behavioral functions of the insect mushroom bodies. Curr Opin Neurobiol..

[evu035-B69] Zha XF (2009). Dosage analysis of Z chromosome genes using microarray in silkworm, *Bombyx mori*. Insect Biochem Mol..

[evu035-B70] Zhan S, Merlin C, Boore JL, Reppert SM (2011). The monarch butterfly genome yields insights into long-distance migration. Cell.

[evu035-B71] Zhang SBO, Mathur S, Hattem G, Tassy O, Pourquie O (2010). Sex-dimorphic gene expression and ineffective dosage compensation of Z-linked genes in gastrulating chicken embryos. BMC Genomics.

[evu035-B72] Zhong S (2011). High-throughput illumina strand-specific RNA sequencing library preparation. Cold Spring Harb Protoc..

