# Supplementary material for: Complete Dosage Compensation and Sex-Biased Gene Expression in the Moth Manduca sexta
Source: Genome Biol Evol. 2014 Feb 19;6(3):526–37. doi: 10.1093/gbe/evu035 (PMC3971586; doi:10.1093/gbe/evu035)
Supplement: Supplementary Data [file supp_6_3_526__index.html]

Complete dosage compensation and sex-biased gene expression in the moth Manduca sexta — Complete Dosage Compensation and Sex-Biased Gene Expression in the Moth Manduca sexta — Supplementary Data 

# Complete Dosage Compensation and Sex-Biased Gene Expression in the Moth *Manduca sexta*

## Supplementary Data

files

**Files in this Data Supplement:**

- Supplementary Data - pdf file
- Supplementary Data - xlsx file
- Supplementary Data - xlsx file
- Supplementary Data - xlsx file
- Supplementary Data - xlsx file
- Supplementary Data - xlsx file
- Supplementary Data - xlsx file
