# Supplementary material for: Complete Dosage Compensation and Sex-Biased Gene Expression in the Moth Manduca sexta
Source: Genome Biol Evol. 2014 Feb 19;6(3):526–37. doi: 10.1093/gbe/evu035 (PMC3971586; doi:10.1093/gbe/evu035)
Supplement: Supplementary Data [file supp_evu035_Supplementary_Material.pdf]

Supplementary figures (S1-S6), tables (S1-S2) & tables S3-S8 headings

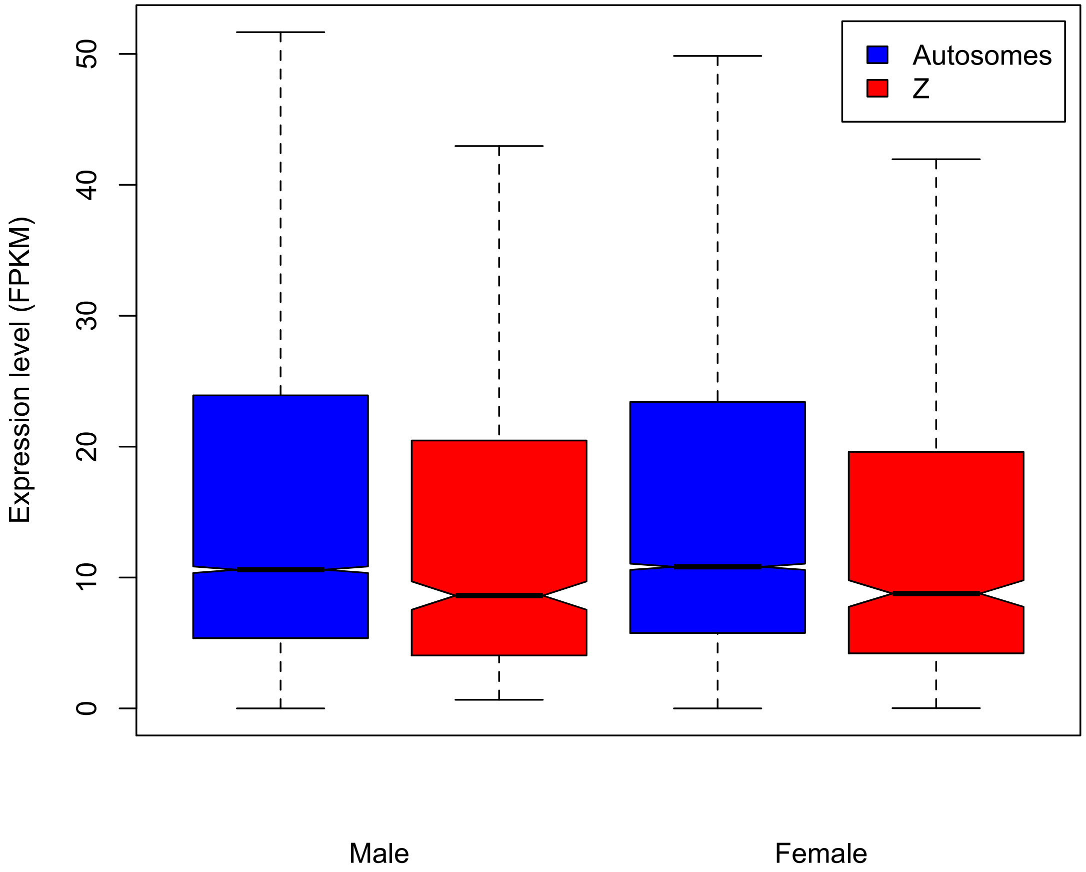

Figure S1
Average Z-linked and autosomal contig expression levels (FPKM) across four replicate males and females for all contigs, unfiltered (for contig *n* see Table 1). Black lines are the median of the FPKM distribution across contigs, boxes show the interquartile range, whiskers extend to 1.5 × the interquartile range and notches approximate the 95% confidence intervals of the medians. Overlapping notches are evidence for the similarity of median values.

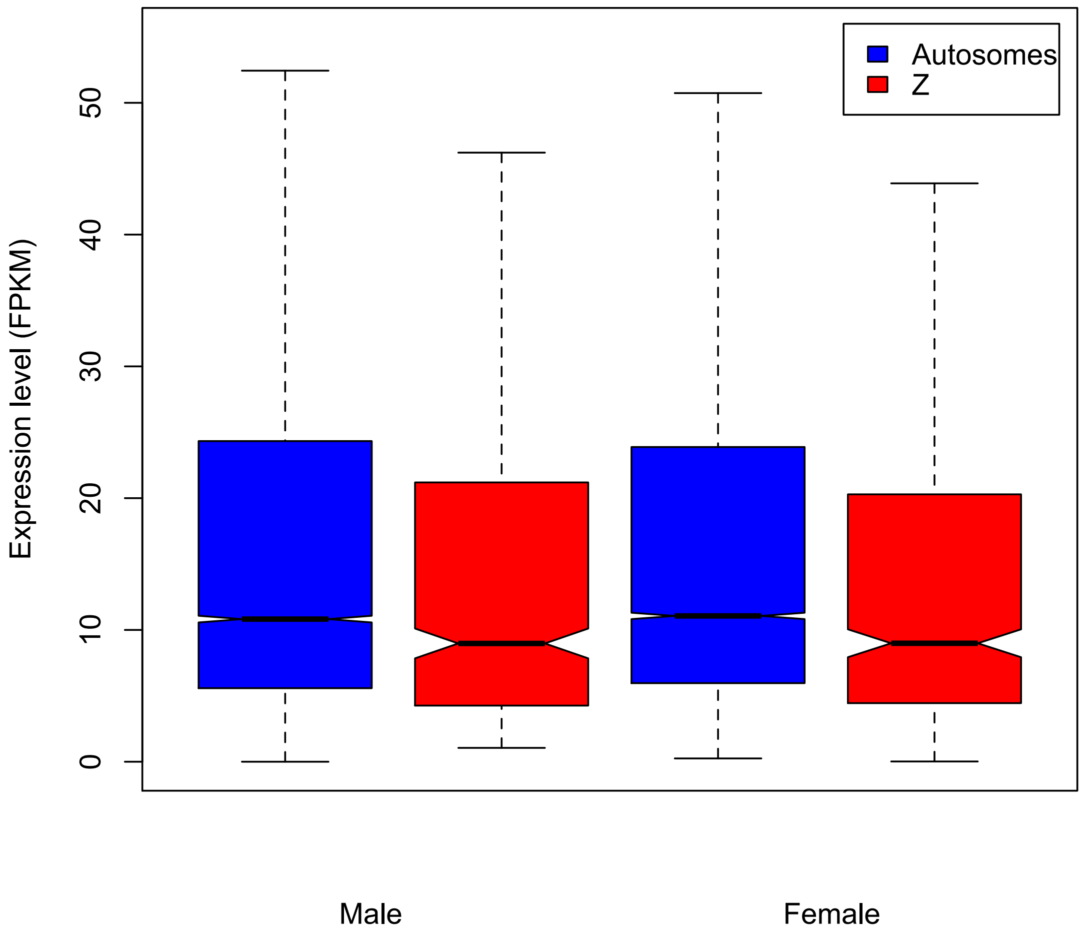

Figure S2

Average Z-linked and autosomal contig expression levels (FPKM) across four replicate males and females, filtering out contigs with <2 FPKM (for contig *n* see Table 1). Black lines are the median of the FPKM distribution across contigs, boxes show the interquartile range, whiskers extend to 1.5 × the interquartile range and notches approximate the 95% confidence intervals of the medians. Overlapping notches are evidence for the similarity of median values.

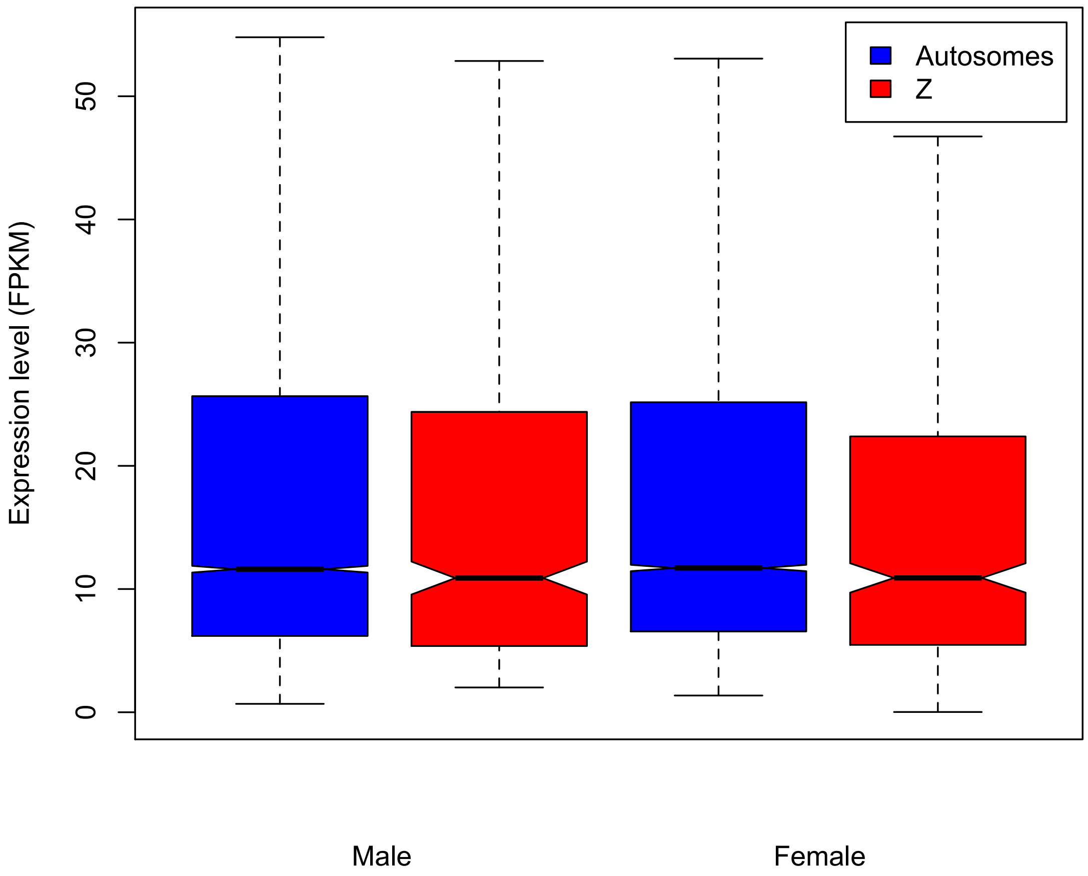

Figure S3

Average Z-linked and autosomal contig expression levels (FPKM) across four replicate males and females, filtering out contigs with <3 FPKM (for contig *n* see Table 1). Black lines are the median of the FPKM distribution across contigs, boxes show the interquartile range, whiskers extend to 1.5 × the interquartile range and notches approximate the 95% confidence intervals of the medians. Overlapping notches are evidence for the similarity of median values.

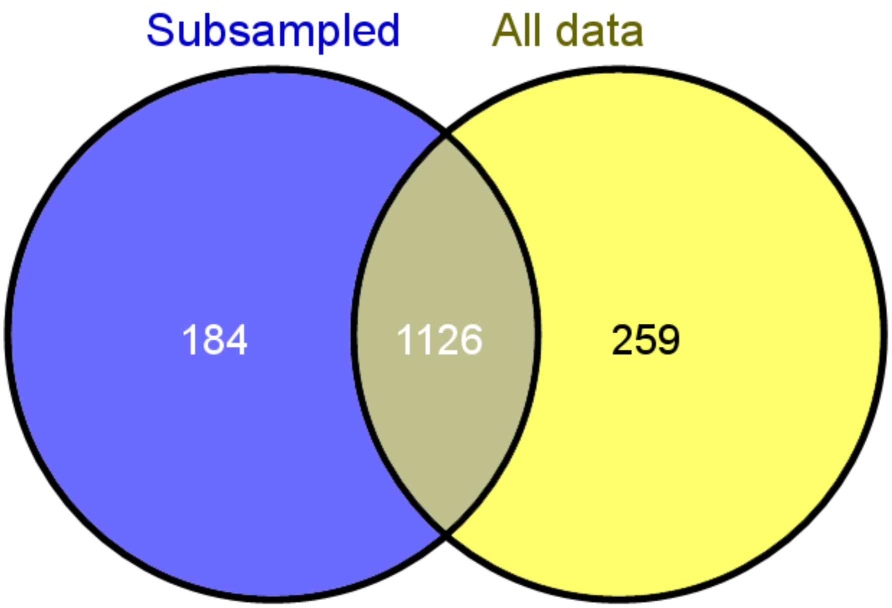

Figure S4

Venn diagram comparing the overlap between significant sex-biased genes (FDR <0.05) from the full dataset libraries (All data) and sub-sampled data set (Subsampled). Venn diagram was constructed in Venny (http://bioinfogp.cnb.csic.es/tools/venny/).

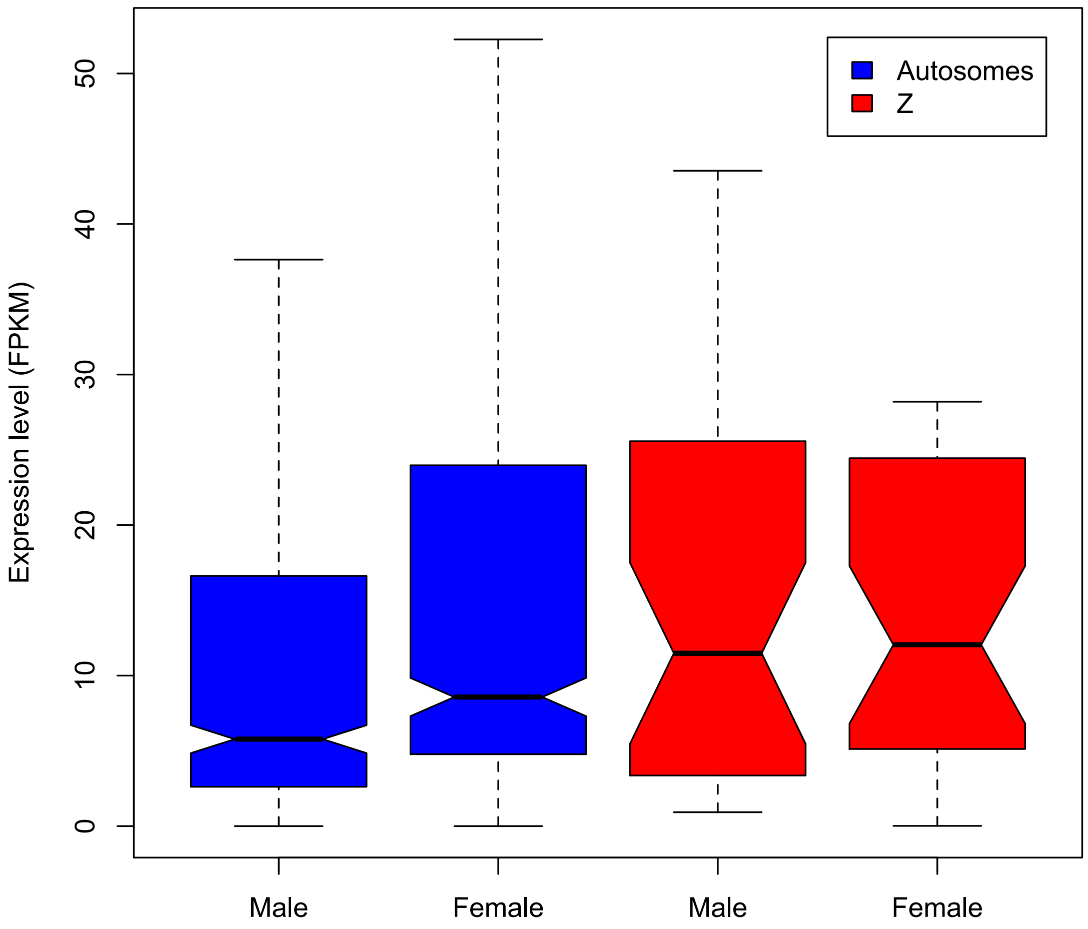

Figure S5
Average Z-linked and autosomal contig expression levels (FPKM) across four replicate males and females for sex-biased genes with putative physical locations (autosomal n = 570, Z n = 34). Physical locations were determined through 1:1 orthologs in *B. mori*. Black lines are the median of the FPKM distribution across contigs, boxes show the interquartile range, whiskers extend to 1.5 × the interquartile range and notches approximate the 95% confidence intervals of the medians. Overlapping notches are evidence for the similarity of median values.

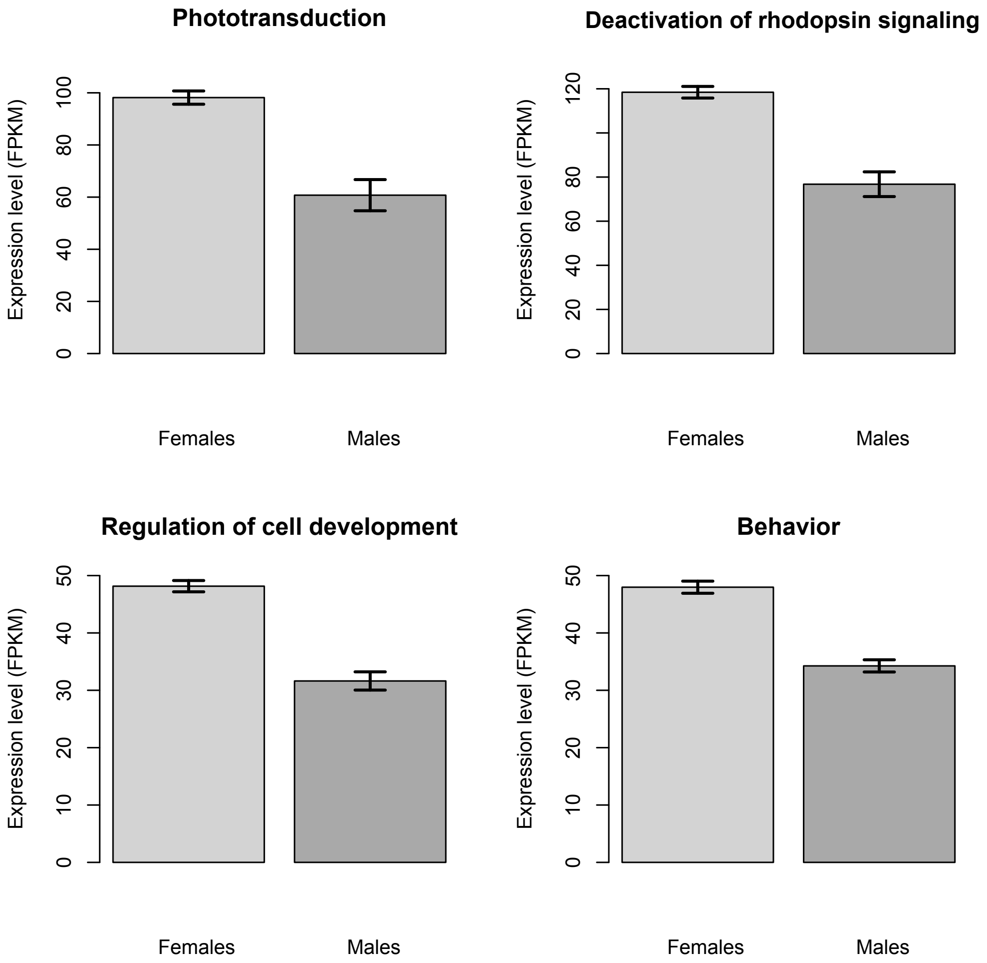

Figure S6
Male and female contig expression levels by function. Contigs were chosen by their inclusion in significantly enriched GO terms in the DAVID functional analysis. Four GO terms were surveyed and expression levels represent average FPKM across four male and four female replicates. GO terms were; Phototransduction (8 genes), Deactivation of rhodopsin signaling (5 genes), Regulation of cell development (11 genes) and Behavior (17 genes). Bars and notches show the standard error.

Table S1

Average expression level of genes, and average expression difference (%), according to chromosomal location (Z or autosome), sex and filtering method. See Table 1 for numbers of genes that contribute to each average expression level.

Filtering method	Z male average FPKM*	Z female average FPKM*
Unfiltered	8.62	8.78
Outlier removal	3.33	3.35
Remove genes <2 FPKM	9	9
Remove genes <3 FPKM	10.89	10.9
Remove genes <4 FPKM	12.52	12.55
	**Autosome male average FPKM***	**Autosome female average FPKM***
Unfiltered	10.6	10.82
Outlier removal	3.6	3.65
Remove genes <2 FPKM	10.82	11.06
Remove genes <3 FPKM	11.61	11.71
Remove genes <4 FPKM	12.7	12.75
	**Z:autosome male % difference**	**Z:autosome female % difference**
Unfiltered	18.7	18.9
Outlier removal	7.5	8.2
Remove genes <2 FPKM	16.8	18.6
Remove genes <3 FPKM	6.2	6.9
Remove genes <4 FPKM	1.4	1.6

***Averages are medians of non-normal distributions and means of normal distributions.**

Table S2

Effects of filtering on the percentage of Z and autosomal contigs removed from each analysis.

Filtering method	Z genes removed	Autosomal genes removed
Unfiltered	0%	0%
Outlier removal	1.2%	0.8%
Remove genes <2 FPKM	2.8%	1.8%
Remove genes <3 FPKM	12.5%	6.7%
Remove genes <4 FPKM	21.6%	13.4%

Spreadsheet table headings

Table S3
Mapping statistics for sub-sampled libraries. Each library was down-sampled to 13 million reads and re-mapped to the contig assembly for differential expression analysis.

Table S4
Full GOrilla functional enrichment test results. GO term IDs and descriptions are provided, along with each GO term *p*-value and FDR, the total number of genes (N), the total number of genes associated with a specific GO term (B), the number of genes in test set (n) and the number of genes in the intersection (b). Enrichment = (b/n) / (B/N).

Table S5
Full DAVID functional enrichment clustering test results. Each cluster includes the enrichment score (the geometric mean of single annotation term *p*-values), the category of annotation term, the term itself with description, the number of genes with that annotation in the test set (Count), gene IDs (Genes), fold enrichment and multiple-test correction statistics (Bonferroni, Benjamini and Hochberg, and FDR).

Table S6
List of significant sex-biased contigs, including *D. melanogaster* orthologs and gene ontology annotations identified through a BLAST search (see Methods).

Table S7
Database of all BLAST hits for *M. sexta* contigs to the *D. melanogaster* genome (see Methods), including *M. sexta* contigs IDs, *D. melanogaster* transcript and gene IDs, and related gene ontology terms.

Table S8
Full results of the differential expression analysis between males and females. Results for each contig (Contig ID) are shown, along with the log fold change (logFC), log counts per million (logCPM), likelihood ratio (LR) and contig-specific *p*-value and FDR.
